# Preliminary Estimates of the Prevalence of Selected Underlying Health Conditions Among Patients with Coronavirus Disease 2019 — United States, February 12–March 28, 2020

**DOI:** 10.15585/mmwr.mm6913e2

**Published:** 2020-04-03

**Authors:** CDC COVID-19 Response Team, CDC COVID-19 Response Team, Nancy Chow, Katherine Fleming-Dutra, Ryan Gierke, Aron Hall, Michelle Hughes, Tamara Pilishvili, Matthew Ritchey, Katherine Roguski, Tami Skoff, Emily Ussery

**Affiliations:** CDC; CDC; CDC; CDC; CDC; CDC; CDC; CDC; CDC; CDC.

On March 11, 2020, the World Health Organization declared Coronavirus Disease 2019 (COVID-19) a pandemic (*1*). As of March 28, 2020, a total of 571,678 confirmed COVID-19 cases and 26,494 deaths have been reported worldwide (*2*). Reports from China and Italy suggest that risk factors for severe disease include older age and the presence of at least one of several underlying health conditions (*3*,*4*). U.S. older adults, including those aged ≥65 years and particularly those aged ≥85 years, also appear to be at higher risk for severe COVID-19–associated outcomes; however, data describing underlying health conditions among U.S. COVID-19 patients have not yet been reported (*5*). As of March 28, 2020, U.S. states and territories have reported 122,653 U.S. COVID-19 cases to CDC, including 7,162 (5.8%) for whom data on underlying health conditions and other known risk factors for severe outcomes from respiratory infections were reported. Among these 7,162 cases, 2,692 (37.6%) patients had one or more underlying health condition or risk factor, and 4,470 (62.4%) had none of these conditions reported. The percentage of COVID-19 patients with at least one underlying health condition or risk factor was higher among those requiring intensive care unit (ICU) admission (358 of 457, 78%) and those requiring hospitalization without ICU admission (732 of 1,037, 71%) than that among those who were not hospitalized (1,388 of 5,143, 27%). The most commonly reported conditions were diabetes mellitus, chronic lung disease, and cardiovascular disease. These preliminary findings suggest that in the United States, persons with underlying health conditions or other recognized risk factors for severe outcomes from respiratory infections appear to be at a higher risk for severe disease from COVID-19 than are persons without these conditions.

Data from laboratory-confirmed COVID-19 cases reported to CDC from 50 states, four U.S. territories and affiliated islands, the District of Columbia, and New York City with February 12–March 28, 2020 onset dates were analyzed. Cases among persons repatriated to the United States from Wuhan, China, and the Diamond Princess cruise ship were excluded. For cases with missing onset dates, date of onset was estimated by subtracting 4 days (median interval from symptom onset to specimen collection date among cases with known dates in these data) from the earliest specimen collection. Public health departments reported cases to CDC using a standardized case report form that captures information (yes, no, or unknown) on the following conditions and potential risk factors: chronic lung disease (inclusive of asthma, chronic obstructive pulmonary disease [COPD], and emphysema); diabetes mellitus; cardiovascular disease; chronic renal disease; chronic liver disease; immunocompromised condition; neurologic disorder, neurodevelopmental, or intellectual disability; pregnancy; current smoking status; former smoking status; or other chronic disease (*6*). Data reported to CDC are preliminary and can be updated by health departments over time; critical data elements might be missing at the time of initial report; thus, this analysis is descriptive, and no statistical comparisons could be made.

The percentages of patients of all ages with underlying health conditions who were not hospitalized, hospitalized without ICU admission, and hospitalized with ICU admission were calculated. Percentages of hospitalizations with and without ICU admission were estimated for persons aged ≥19 years with and without underlying health conditions. This part of the analysis was limited to persons aged ≥19 years because of the small sample size of cases in children with reported underlying health conditions (N = 32). To account for missing data among these preliminary reports, ranges were estimated with a lower bound including cases with both known and unknown status for hospitalization with and without ICU admission as the denominator and an upper bound using only cases with known outcome status as the denominator. Because of small sample size and missing data on underlying health conditions among COVID-19 patients who died, case-fatality rates for persons with and without underlying conditions were not estimated.

As of March 28, 2020, a total of 122,653 laboratory-confirmed COVID-19 cases (Figure) and 2,112 deaths were reported to CDC. Case report forms were submitted to CDC for 74,439 (60.7%) cases. Data on presence or absence of underlying health conditions and other recognized risk factors for severe outcomes from respiratory infections (i.e., smoking and pregnancy) were available for 7,162 (5.8%) patients (Table 1). Approximately one third of these patients (2,692, 37.6%), had at least one underlying condition or risk factor. Diabetes mellitus (784, 10.9%), chronic lung disease (656, 9.2%), and cardiovascular disease (647, 9.0%) were the most frequently reported conditions among all cases. Among 457 ICU admissions and 1,037 non-ICU hospitalizations, 358 (78%) and 732 (71%), respectively occurred among persons with one or more reported underlying health condition. In contrast, 1,388 of 5,143 (27%) COVID-19 patients who were not hospitalized were reported to have at least one underlying health condition.

**FIGURE:**
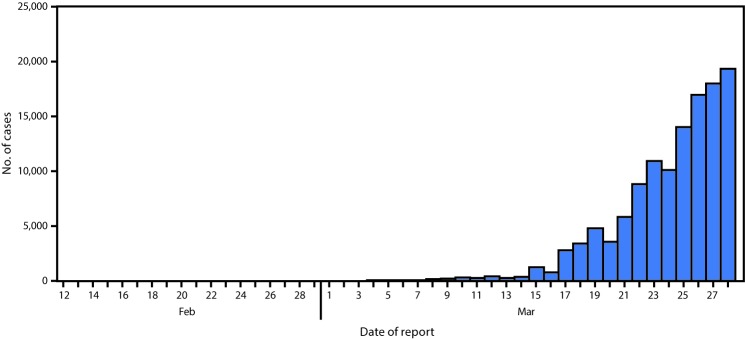
Daily number of reported COVID-19 cases* — United States, February 12–March 28, 2020^†^ * Cases among persons repatriated to the United States from Wuhan, China, and the Diamond Princess cruise ship are excluded. ^†^ Cumulative number of COVID-19 cases reported daily by jurisdictions to CDC using aggregate case count was 122,653 through March 28, 2020.

**TABLE 1:** Reported outcomes among COVID-19 patients of all ages, by hospitalization status, underlying health condition, and risk factor for severe outcome from respiratory infection — United States, February 12–March 28, 2020

Underlying health condition/Risk factor for severe outcomes from respiratory infection (no., % with condition)	No. (%)			
	Not hospitalized	Hospitalized, non-ICU	ICU admission	Hospitalization status unknown
**Total with case report form (N = 74,439)**	**12,217**	**5,285**	**1,069**	**55,868**
**Missing or unknown status for all conditions (67,277)**	**7,074**	**4,248**	**612**	**55,343**
**Total with completed information (7,162)**	**5,143**	**1,037**	**457**	**525**
One or more conditions (2,692, 37.6%)	1,388 (27)	732 (71)	358 (78)	214 (41)
Diabetes mellitus (784, 10.9%)	331 (6)	251 (24)	148 (32)	54 (10)
Chronic lung disease* (656, 9.2%)	363 (7)	152 (15)	94 (21)	47 (9)
Cardiovascular disease (647, 9.0%)	239 (5)	242 (23)	132 (29)	34 (6)
Immunocompromised condition (264, 3.7%)	141 (3)	63 (6)	41 (9)	19 (4)
Chronic renal disease (213, 3.0%)	51 (1)	95 (9)	56 (12)	11 (2)
Pregnancy (143, 2.0%)	72 (1)	31 (3)	4 (1)	36 (7)
Neurologic disorder, neurodevelopmental, intellectual disability (52, 0.7%)^†^	17 (0.3)	25 (2)	7 (2)	3 (1)
Chronic liver disease (41, 0.6%)	24 (1)	9 (1)	7 (2)	1 (0.2)
Other chronic disease (1,182, 16.5%)^§^	583 (11)	359 (35)	170 (37)	70 (13)
Former smoker (165, 2.3%)	80 (2)	45 (4)	33 (7)	7 (1)
Current smoker (96, 1.3%)	61 (1)	22 (2)	5 (1)	8 (2)
None of the above conditions^¶^ (4,470, 62.4%)	3,755 (73)	305 (29)	99 (22)	311 (59)

**Abbreviation:** ICU = intensive care unit.

* Includes any of the following: asthma, chronic obstructive pulmonary disease, and emphysema.

^†^ For neurologic disorder, neurodevelopmental, and intellectual disability, the following information was specified: dementia, memory loss, or Alzheimer’s disease (17); seizure disorder (5); Parkinson’s disease (4); migraine/headache (4); stroke (3); autism (2); aneurysm (2); multiple sclerosis (2); neuropathy (2); hereditary spastic paraplegia (1); myasthenia gravis (1); intracranial hemorrhage (1); and altered mental status (1).

^§^ For other chronic disease, the following information was specified: hypertension (113); thyroid disease (37); gastrointestinal disorder (32); hyperlipidemia (29); cancer or history of cancer (29); rheumatologic disorder (19); hematologic disorder (17); obesity (17); arthritis, nonrheumatoid, including not otherwise specified (16); musculoskeletal disorder other than arthritis (10); mental health condition (9); urologic disorder (7); cerebrovascular disease (7); obstructive sleep apnea (7); fibromyalgia (7); gynecologic disorder (6); embolism, pulmonary or venous (5); ophthalmic disorder (2); hypertriglyceridemia (1); endocrine (1); substance abuse disorder (1); dermatologic disorder (1); genetic disorder (1).

^¶^ All listed chronic conditions, including other chronic disease, were marked as not present.

Among patients aged ≥19 years, the percentage of non-ICU hospitalizations was higher among those with underlying health conditions (27.3%–29.8%) than among those without underlying health conditions (7.2%–7.8%); the percentage of cases that resulted in an ICU admission was also higher for those with underlying health conditions (13.3%–14.5%) than those without these conditions (2.2%–2.4%) (Table 2). Small numbers of COVID-19 patients aged <19 years were reported to be hospitalized (48) or admitted to an ICU (eight). In contrast, 335 patients aged <19 years were not hospitalized and 1,342 had missing data on hospitalization. Among all COVID-19 patients with complete information on underlying conditions or risk factors, 184 deaths occurred (all among patients aged ≥19 years); 173 deaths (94%) were reported among patients with at least one underlying condition. 

**TABLE 2:** Hospitalization with and without intensive care unit (ICU) admission, by age group among COVID-19 patients aged ≥19 years with and without reported underlying health conditions — United States, February 12–March 28, 2020*

Age group (yrs)	Hospitalized without ICU admission, No. (% range^†^)		ICU admission, No. (% range^†^)	
	Underlying condition present/reported^§^		Underlying condition present/reported^§^	
	Yes	No	Yes	No
19–64	285 (18.1–19.9)	197 (6.2–6.7)	134 (8.5–9.4)	58 (1.8–2.0)
≥65	425 (41.7–44.5)	58 (16.8–18.3)	212 (20.8–22.2)	20 (5.8–6.3)
**Total ≥19**	**710 (27.3–29.8)**	**255 (7.2–7.8)**	**346 (13.3–14.5)**	**78 (2.2–2.4)**

* Includes COVID-19 patients aged ≥19 years with known status on underlying conditions.

^†^ Lower bound of range = number of persons hospitalized or admitted to an ICU among total in row stratum; upper bound of range = number of persons hospitalized or admitted to an ICU among total in row stratum with known outcome status: hospitalization or ICU admission status.

**^§^** Includes any of following underlying health conditions or risk factors: chronic lung disease (including asthma, chronic obstructive pulmonary disease, and emphysema); diabetes mellitus; cardiovascular disease; chronic renal disease; chronic liver disease; immunocompromised condition; neurologic disorder, neurodevelopmental, or intellectual disability; pregnancy; current smoker; former smoker; or other chronic disease.

## Discussion

Among 122,653 U.S. COVID-19 cases reported to CDC as of March 28, 2020, 7,162 (5.8%) patients had data available pertaining to underlying health conditions or potential risk factors; among these patients, higher percentages of patients with underlying conditions were admitted to the hospital and to an ICU than patients without reported underlying conditions. These results are consistent with findings from China and Italy, which suggest that patients with underlying health conditions and risk factors, including, but not limited to, diabetes mellitus, hypertension, COPD, coronary artery disease, cerebrovascular disease, chronic renal disease, and smoking, might be at higher risk for severe disease or death from COVID-19 (*3*,*4*). This analysis was limited by small numbers and missing data because of the burden placed on reporting health departments with rapidly rising case counts, and these findings might change as additional data become available.

It is not yet known whether the severity or level of control of underlying health conditions affects the risk for severe disease associated with COVID-19. Many of these underlying health conditions are common in the United States: based on self-reported 2018 data, the prevalence of diagnosed diabetes among U.S. adults was 10.1% (*7*), and the U.S. age-adjusted prevalence of all types of heart disease (excluding hypertension without other heart disease) was 10.6% in 2017 (*8*). The age-adjusted prevalence of COPD among U.S. adults is 5.9% (*9*), and in 2018, the U.S. estimated prevalence of current asthma among persons of all ages was 7.9% (*7*). CDC continues to develop and update resources for persons with underlying health conditions to reduce the risk of acquiring COVID-19 (*10*). The estimated higher prevalence of these conditions among those in this early group of U.S. COVID-19 patients and the potentially higher risk for more severe disease from COVID-19 associated with the presence of underlying conditions highlight the importance of COVID-19 prevention in persons with underlying conditions.

The findings in this report are subject to at least six limitations. First, these data are preliminary, and the analysis was limited by missing data related to the health department reporting burden associated with rapidly rising case counts and delays in completion of information requiring medical chart review; these findings might change as additional data become available. Information on underlying conditions was only available for 7,162 (5.8%) of 122,653 cases reported to CDC. It cannot be assumed that those with missing information are similar to those with data on either hospitalizations or underlying health conditions. Second, these data are subject to bias in outcome ascertainment because of short follow-up time. Some outcomes might be underestimated, and long-term outcomes cannot be assessed in this analysis. Third, because of the limited availability of testing in many jurisdictions during this period, this analysis is likely biased toward more severe cases, and findings might change as testing becomes more widespread. Fourth, because of the descriptive nature of these data, attack rates among persons with and without underlying health conditions could not be compared, and thus the risk difference of severe disease with COVID-19 between these groups could not be estimated. Fifth, no conclusions could be drawn about underlying conditions that were not included in the case report form or about different conditions that were reported in a single, umbrella category. For example, asthma and COPD were included in a chronic lung disease category. Finally, for some underlying health conditions and risk factors, including neurologic disorders, chronic liver disease, being a current smoker, and pregnancy, few severe outcomes were reported; therefore, conclusions cannot be drawn about the risk for severe COVID-19 among persons in these groups.

Persons in the United States with underlying health conditions appear to be at higher risk for more severe COVID-19, consistent with findings from other countries. Persons with underlying health conditions who have symptoms of COVID-19, including fever, cough, or shortness of breath, should immediately contact their health care provider. These persons should take steps to protect themselves from COVID-19, through washing their hands; cleaning and disinfecting high-touch surfaces; and social distancing, including staying at home, avoiding crowds, gatherings, and travel, and avoiding contact with persons who are ill. Maintaining at least a 30-day supply of medication, a 2-week supply of food and other necessities, and knowledge of COVID-19 symptoms are recommended for those with underlying health conditions (*10*). All persons should take steps to protect themselves from COVID-19 and to protect others. All persons who are ill should stay home, except to get medical care; should not go to work; and should stay away from others. This is especially important for those who work with persons with underlying conditions or who otherwise are at high risk for severe outcomes from COVID-19. Community mitigation strategies, which aim to slow the spread of COVID-19, are important to protect all persons from COVID-19, especially persons with underlying health conditions and other persons at risk for severe COVID-19–associated disease (https://www.cdc.gov/coronavirus/2019-ncov/downloads/community-mitigation-strategy.pdf).

**Summary**

**What is already known about this topic?**

Published reports from China and Italy suggest that risk factors for severe COVID-19 disease include underlying health conditions, but data describing underlying health conditions among U.S. COVID-19 patients have not yet been reported.

**What is added by this report?**

Based on preliminary U.S. data, persons with underlying health conditions such as diabetes mellitus, chronic lung disease, and cardiovascular disease, appear to be at higher risk for severe COVID-19–associated disease than persons without these conditions.

**What are the implications for public health practice?**

Strategies to protect all persons and especially those with underlying health conditions, including social distancing and handwashing, should be implemented by all communities and all persons to help slow the spread of COVID-19.
